# The Composition and Biological Activity of Honey: A Focus on Manuka Honey

**DOI:** 10.3390/foods3030420

**Published:** 2014-07-21

**Authors:** José M. Alvarez-Suarez, Massimiliano Gasparrini, Tamara Y. Forbes-Hernández, Luca Mazzoni, Francesca Giampieri

**Affiliations:** 1 Department of Odontostomatologic and Specialized Clinical Sciences, Faculty of Medicine and Surgery, Polytechnic University of Marche, Avenue Ranieri 65, Ancona 60100, Italy; E-Mails: m.gasparrini@univpm.it (M.G.); tamara.forbe@gmail.com (T.Y.F.-H.); l.mazzoni@univpm.it (L.M.); 2 Department of Nutrition and Health, International Iberoamerican University (UNINI), Avenue Adolfo Ruiz Cortines 112, Torres de Cristal L 101 A-3, Campeche 24040, México; 3 Department of Agricultural, Food and Environmental Sciences, Polytechnic University of Marche, Via Ranieri 65, Ancona 60100, Italy

**Keywords:** manuka honey, polyphenolic composition, wound treatments, antimicrobial activity

## Abstract

Honey has been used as a food and medical product since the earliest times. It has been used in many cultures for its medicinal properties, as a remedy for burns, cataracts, ulcers and wound healing, because it exerts a soothing effect when initially applied to open wounds. Depending on its origin, honey can be classified in different categories among which, monofloral honey seems to be the most promising and interesting as a natural remedy. Manuka honey, a monofloral honey derived from the manuka tree (*Leptospermum scoparium*), has greatly attracted the attention of researchers for its biological properties, especially its antimicrobial and antioxidant capacities. Our manuscript reviews the chemical composition and the variety of beneficial nutritional and health effects of manuka honey. Firstly, the chemical composition of manuka honey is described, with special attention given to its polyphenolic composition and other bioactive compounds, such as glyoxal and methylglyoxal. Then, the effect of manuka honey in wound treatment is described, as well as its antioxidant activity and other important biological effects.

## 1. Introduction

Honey is a sweet and flavorful natural product, which is consumed for its high nutritive value and for its effects on human health, with antioxidant, bacteriostatic, anti-inflammatory and antimicrobial properties, as well as wound and sunburn healing effects [1]. Honey is produced by bees from plant nectars, plant secretions and excretions of plant-sucking insects. Concerning its nutrient profile, it represents an interesting source of natural macro- and micro-nutrients, consisting of a saturated solution of sugars, of which fructose and glucose are the main contributors, but also of a wide range of minor constituents, especially phenolic compounds [2,3]. The composition of honey is rather variable and depends primarily on its floral source; seasonal and environmental factors can also influence its composition and its biological effects. Several studies have shown that the antioxidant potential of honey is strongly correlated not only with the concentration of total phenolics present, but also with the color, with dark colored honeys being reported to have higher total phenolic contents and, consequently, higher antioxidant capacities [3,4,5,6].

According to the origin, honey can be classified in different categories as follows: (1) blossom honey, obtained predominantly from the nectar of flowers (as opposed to honeydew honey); (2) honeydew honey, produced by bees after they collect “honeydew” (secretions of insects belonging to the genus, *Rhynchota*), which pierce plant cells, ingest plant sap and then secrete it again; (3) monofloral honey, in which the bees forage predominantly on one type of plant and which is named according to the plant; and (4) multifloral honey (also known as polyfloral) that has several botanical sources, none of which is predominant, e.g., meadow blossom honey and forest honey.

It is has been suggested that many of the medicinal properties of plants can be transmitted through honey, so that honey could be used as a vehicle for transporting plant medicinal properties [3]. Within monofloral honey, manuka honey, a dark honey, has greatly attracted the attention of the international scientific community for its biological properties, especially for its antimicrobial and antioxidant capacities. This honey is derived from the manuka tree, *Leptospermum scoparium*, of the Myrtaceae family, which grows as a shrub or a small tree throughout New Zealand and eastern Australia [7]. In traditional medicine, different extracts of the manuka tree are used as sedatives and wound-healing remedies. Moreover, manuka honey itself has long been employed for clearing up infections, including abscesses, surgical wounds, traumatic wounds, burns and ulcers of different etiology [8]. Currently, the main bioactive compounds in manuka honey and the mechanisms responsible for their biological activities are being studied. These studies would support the increased use of manuka honey in skin medicine, and they can also be the basis for the isolation and purification of compounds for the development of bio-pharmaceutical products with antimicrobial properties and wound healing properties; these new findings could represent an added economic value that can favor also the beekeepers in their productions.

This review focuses on the phytochemical composition of manuka honey and on its biological effects. An overview of the most abundant phytochemicals is presented, with particular attention to recent evidence on its antimicrobial activity and its impact on wound treatments, as well as on its antioxidant capacity.

## 2. Chemical Composition

Polyphenolic characterization has proven to be suitable for the differentiation of the floral origin of honeys [9], and therefore, flavonoids could represent a valid botanical marker for honey [10], being closely related with their antioxidant capacity. The qualitative and quantitative difference in flavonoid contents of manuka honey determined in diverse studies may represent the result of the different extraction and detection methods applied, and this limit makes the data available in the literature difficult to compare. The major compounds identified are represented in Table 1. Several studies have determined that the major flavonoids in manuka honey are: pinobanksin, pinocembrin and chrysin, while luteolin, quercetin, 8-methoxykaempferol, isorhamnetin, kaempferol and galangin have been also identified in minor concentration [11,12,13].

Regarding phenolic acids and volatile norisoprenoids constituents, Oelschlaegel *et al*. [13] detected different profiles in manuka honey attributed to three chemotypes of *L. scoparium* in New Zealand. The first group was characterized by high levels of 4-hydroxybenzoic acid, dehydrovomifoliol and benzoic acid yields, the second one by high concentrations of kojic acid and 2-methoxybenzoic acid and the third group by high contents of syringic acid, 4-methoxyphenyllactic acid and methyl syringate. According to the determined average amounts, phenylacetic acid, phenyllactic acid, 4-methoxyphenyllactic acid, leptosin and methyl syringate were the dominating compounds [14,15]. 

**Figure 1:**
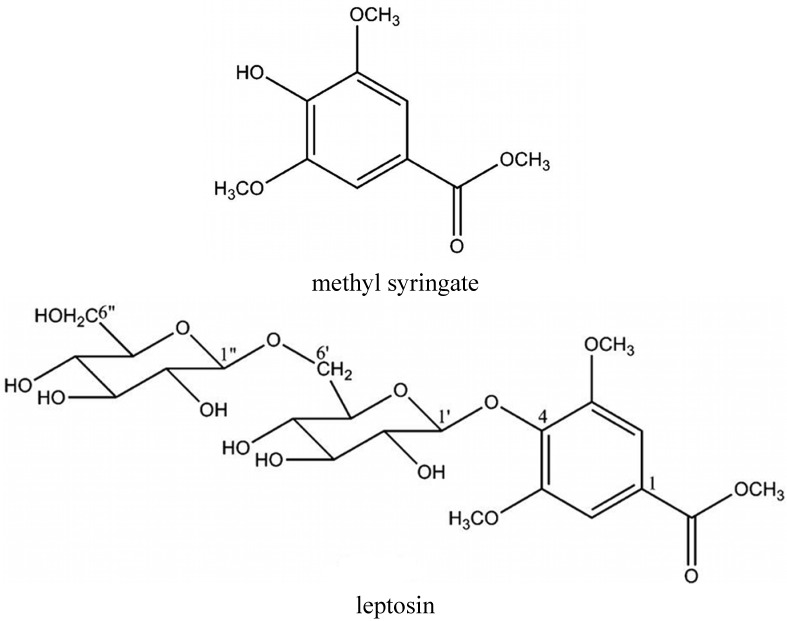
Chemical structures of methyl syringate and leptosin.

Methyl syringate (MSYR) and leptosin (the novel glycoside of MSYR, methyl syringate 4-*O*-β-d-gentiobiose) (Figure 1) are the active compounds from manuka honey to which its myeloperoxidase (MPO)-activity inhibition is ascribed. Although the biological activities and biosynthetic pathway/origin of the glycoside are still unknown, it may be a good chemical marker for the purity of manuka honey [7].

Other constituents of interest found in manuka honey are: different 1,2-dicarbonyl compounds, such as glyoxal (GO), 3-deoxyglucosulose (3-DG) and methylglyoxal (MGO). These compounds are typically formed during the Maillard reaction or caramelization reactions as degradation products from reducing carbohydrates, and they have been identified as important contributors to the non-peroxide antibacterial activity [13,16,17].

From the nutritional point of view, the physiological significance resulting from the uptake of MGO and other 1,2-dicarbonyl compounds must be a topic of further investigations. MGO and glycation compounds resulting from the reaction of MGO with amino acid side chains of lysine or arginine, respectively, have been identified *in vivo* and are associated with complications of diabetes and some neurodegenerative diseases, although the role of these compounds in the pathogenesis of different diseases have not yet been fully understood [16].

**Table 1:** Most common compounds identified in manuka honey.

Phenolic Acid and Flavonoids	Ref.	Other Compounds	Ref.
Caffeic acid	[12,13]	Phenyllactic acid	[13]
Isoferulic acid	[12]	4-Methoxyphenolactic acid	[13]
*p*-Coumaric acid	[12]	Kojic acid	[13]
Gallic acid	[13,17]	5-Hydroxymethylfurfural	[13]
4-Hydrobenzoic acid	[13]	2-Methoxybenzoic acid	[13]
Syringin acid	[13]	Phenylacetic acid	[13]
Quercetin	[12,17]	Methyl syringate	[13]
Luteolin	[12,13]	Dehydrovomifoliol	[13]
8-Methoxykaempferol	[12]	Leptosin	[13]
Pinocembrin	[12]	Glyoxal	[13,16]
Isorhamnetin	[12,17]	Methylglyoxal	[13,16]
Kaempferol	[12]	3-Deoxyglucosulose	[13,16]
Chrysin	[12]	-	-
Galangin	[12]	-	-
Pinobanksin	[12]	-	-

## 3. Use of Manuka Honey in Wound Treatments

The importance of honey in the field of wound treatments has been well known since ancient times. This healing property is related to the antioxidant and antibacterial activity that honey offers, maintaining a moist wound condition, and to the high viscosity that provides a protective barrier on the wound, preventing microbial infection. Its immunological activity is relevant also for wound repair, exerting the same time pro- and anti-inflammatory effects [18,19,20,21,22,23]. Normal wound healing is a complex process composed of a series of overlapping events (coagulation, inflammation, cell proliferation, tissue remodeling) in which the damaged tissue is gradually removed and replaced by restorative tissues [24]. While normal inflammation resolves within 1–2 days as the neutrophil number decreases, the accumulation of these cells in the wound site contributes to a disordered network of regulatory cytokines, leaving the wound in a chronic state of inflammation [25]. In these chronic wounds, bacterial cells predominantly exist as biofilms, where cells are embedded within a matrix of polysaccharides and other components that limit the availability of antibiotics for wound healing. Furthermore, the emergence of bacterial resistance to multiple antibiotics has worsened the problem of chronic wound biofilm treatment [26].

Current therapeutic products widely used in wound care (silver sulfadiazine (SSD), hydrogel, hydrocolloid and alginate dressings impregnated with silver) are considered useful for limiting bacterial infections, even if excessive use of ionic silver has generated some concern regarding the development of bacterial resistance [27,28]; this situation, in recent years, has stimulated modern medicine to focus attention on natural products with antimicrobial activity and their use in clinical practice. The low cost and absence of the antimicrobial resistance risk of natural products, such as honey, aloe vera or curcumin, are the major arguments for implementing natural products in wound treatment [29]. Although it is an ancient topical treatment for wounds, honey has been currently established in conventional medicine as a licensed medical device, either combined into sterile dressings or sterilized in tubes [30].

The healing time decrease after honey treatment can be explained through a dual effect on the inflammatory response. Firstly, honey prevents a prolonged inflammatory response suppressing the production and propagation of inflammatory cells at the wound site; secondly, it stimulates the production of proinflammatory cytokine, allowing normal healing to occur [31] and stimulating the proliferation of fibroblasts and epithelial cells [32,33]. The effect of honey and its components on the production of inflammatory cytokines has been evaluated in primary human monocytes cells [34]. In these studies, it was shown that manuka honey stimulated the production of inflammatory cytokines TNF-α, IL-1β or IL-6 via a TLR4-dependent mechanism. For the first time, a 5.8-kDa component responsible for cytokine induction in human monocytes via TLR4 was isolated from manuka honey [35].

Microorganisms that colonize a burn wound originate from the patient’s gastrointestinal and respiratory flora, from endogenous skin or from contaminated external sources (soil, water, air) [36]. The topical application of honey rapidly clears wound infection, promoting the healing process of deep surgical infected wounds [37,38,39], also when they do not respond to conventional antibiotic and antiseptic therapy [37]. Furthermore, in burn wounds, honey application decreases the wound area, exerts an antibacterial effect and promotes better re-epithelialization compared to hydrofiber silver or SSD treatment. Moreover, the anti-inflammatory action of honey decreases damage caused by free radicals that result from inflammation, preventing further necrosis [40].

**Figure 2:**
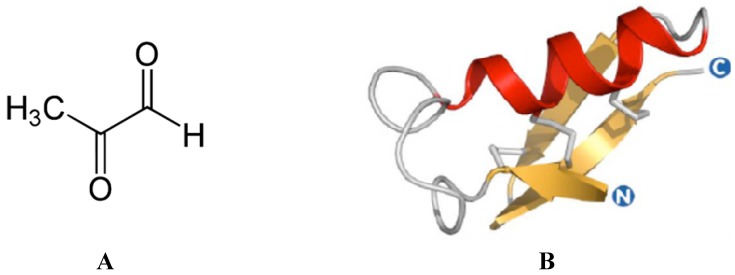
(**A**) Chemical structures of the methylglyoxal (MGO). (**B**) Homology model of defensin-1 from *Apis mellifera*. The model of the mature protein (residues 44–94) was obtained using the experimentally-resolved structure of lucifensin from *Lucilia sericata* (PDB ID: 2LLD) as a template. Alignment and modeling was performed using the Swiss Model server [41]; the figure has been obtained through PyMOL Molecular Graphics System, Version 1.5.0.4, Schrödinger, LLC (Portland, OR, USA).

The antibacterial nature of honey depends on different factors acting singularly or synergistically, the most salient of which are phenolic compounds, wound pH, H_2_O_2_, pH of honey and osmotic pressure exerted by the honey itself [3]. It has been documented that the pronounced antibacterial activity of manuka honey directly originates from the MGO it contains (Figure 2A) [16]. This non-peroxide antibacterial activity due to the presence of MGO is called the unique manuka factor (UMF) [16].

Other antimicrobial compounds in honeys include bee defensin-1 (Figure 2B), various phenolic compounds and complex carbohydrates [1,2]. The combination of these diverse assaults may account for the inability of bacteria to develop resistance to honey, in contrast to the rapid induction of resistance observed with conventional single-component antibiotics [42,43]. A few studies have examined the antimicrobial effect of manuka honey, showing that it is active against a range of bacteria, including Group A *Streptococcus pyogenes*, *Streptococcus mutans*, *Proteus mirabilis*, *Pseudomonas aeruginosa*, *Enterobacter cloacae* and *Staphylococcus aureus* [44,45,46,47]. A list of microorganisms that have been found to be sensitive to manuka honeys is shown in Table 2. Furthermore, no resistant bacteria (*Escherichia coli*, MRSA, *Pseudomonas aeruginosa* and *Staphylococcus epidermidis*) have been isolated after exposure of wound isolates to sub-inhibitory concentrations of manuka honey [42,43]. This seems to be very likely due, at least in part, to differences in the levels of the principle antibacterial components in the honey, MGO and hydrogen peroxide, which varies with the floral and geographic source of nectar, honey storage time and conditions and any other possible treatment that could affect it. Anti-biofilm activity was highest in the honey blend that contained the highest level of manuka-derived honey; the effectiveness of the different manuka-type honeys tested increased with MGO content, although the same level of MGO, with or without sugar, could not eradicate biofilms. This suggests that additional factors in these manuka-type honeys are responsible for their potent anti-biofilm activity [48].

**Table 2:** List of microorganisms that have been found to be sensitive to manuka honeys [49].

Gram Positive Strains	Gram Negative Strains
*Streptococcus pyogenes*	*Stenotrophomonas maltophilia*
Coagulase negative staphylococci	*Acinetobacter baumannii*
Methicillin-resistant *Staphylococcus aureus (*MRSA*)*	*Salmonella enterica* serovar *typhi*
*Streptococcus agalactiae*	*Pseudomonas aeruginosa*
*Staphylococcus aureus*	*Proteus mirabilis*
Coagulase-negative *Staphylococcus aureus (*CONS*)*	*Shigella flexneri*
*Hemolytic streptococci*	*Escherichia coli*
*Enterococcus*	*Enterobacter cloacae*
*Streptococcus mutans*	*Shigella sonnei*
*Streptococcus sobrinus*	*Salmonella typhi*
*Actinomyces viscosus*	*Klebsiella pneumonia*
-	*Stenotrophomonas maltophilia*
-	*Burkholderia cepacia*
-	*Helicobacter pylori*
-	*Campylobacter* spp*.*
-	*Porphyromonas gingivalis*

Manuka honey has been shown to eradicate methicillin-resistant *Staphylococcus aureus* (MRSA) from colonized wounds and to inhibit MRSA *in vitro* by interrupting cell division. Furthermore, the presence of manuka honey restores MRSA susceptibility to oxacillin; molecular analysis indicated that it also affects the regulation of the mecR1 gene, possibly accounting for the restored susceptibility [30]. In another study, a synergistic effect between rifampicin and commercially available FDA-approved manuka honey (Medihoney, Medihoney Ltd, Slough, United Kingdom) was demonstrated on clinical *S. aureus* isolates, including MRSA strains. Unlike with rifampicin alone, in which resistance was observed after overnight incubation on plates, the combination of Medihoney and rifampicin maintained the susceptibility of *S. aureus* to rifampicin [50]. Manuka honey, therefore, seems to offer real potential in providing novel synergistic combinations with antibiotics for treating wound infections of multidrug-resistant (MDR) bacteria. It is interesting to note that the antibiotics that have shown synergy with manuka honey are from different antibiotic classes, which inhibit distinct targets, such as the 30 S ribosome, RNA polymerase, membranes and penicillin binding proteins. This finding supports the idea that honey is a complex substance, perhaps with multiple active components that affect more than one cellular target site [30].

Manuka honey is also known to have a relatively low pH (3.5–4.5), which, besides inhibiting microbial growth, stimulates the bactericidal actions of macrophages and, in chronic wounds, reduces protease activity and increases fibroblast activity and oxygenation [51,52,53]. Growth factors, such as TGF-β, are known to become physiologically active when subjected to an acid treatment, and the use of Medihoney demonstrates a further increase in cellular activity. This impact has been reported in the hDF-based studies and in an *in vitro* wound healing assay study, where Medihoney supplements resulted in statistically significant increases in cell proliferation and migration [25]. 

Finally, manuka honey has been shown to specifically decrease the inflammatory response associated with ulcerative colitis, an inflammatory intestine disease characterized by an overexpression of inflammatory cells, in embryonic kidney cell lines. The anti-inflammatory effect by the manuka honey was strongest in the presence of the Pam3CSK4 ligand, indicating that the honeys act through the TLR1/TLR2 signaling pathway. The anti-inflammatory activity of manuka honeys is therefore pathway specific [31].

## 4. Antioxidant Activity

In addition to antibacterial activity, honeys are known to possess strong antioxidant capacity, which acts in modulating free radical production, thus protecting cell components from their harmful action [54,55].

Manuka honey contains a high amount of phenolic compounds [14,15], as well as other phenolic compounds that have been identified with a potent capacity to reduce free radicals, providing a relevant antioxidant capacity [56,57]. For its relevant bioactive properties, it has often been used in different studies as the “gold standard” [8] to test and evaluate the antioxidant capacity of different kinds of honey from different botanical and geographical origins. Manuka honey, in fact, exhibits the highest value in terms of phenolic content and antioxidant capacity, for example compared to acacia, wild carrot and Portobello honeys [58,59], obtained, respectively, from Germany, Algeria, Saudi Arabia and Scotland. Similar results are obtained with Malaysian monofloral honeys [56] and Tualang honey, a Malaysian multifloral jungle honey [60]. The scavenger role of manuka honey against superoxide anion radicals has also been investigated through electronic paramagnetic resonance [54,61]; the results proved that the quenching properties of manuka honey could be attributed to methyl syringate [62]. Finally, manuka honey seems to exert a protective role against oxidative damage also in an *in vivo* model [57], reducing DNA damage, the malondialdehyde level and glutathione peroxidase activity in the liver of both young and middle-aged groups of rats. These effects could be mediated through the modulation of antioxidant enzyme activities (such as catalase) and through the high antioxidant capacity of its relevant total phenolic content. The results obtained suggest a possible use of manuka honey as an alternative natural supplement to improve the physiological oxidative status.

## 5. Other Effects

In addition to its antimicrobial and antioxidant activities, recent studies demonstrated that honey can exert anti-proliferative effects against cancer cells [62,63,64]. These anticancer properties can involve different processes: (1) the apoptosis of cancer cells through the depolarization of the mitochondrial membrane, (2) the inhibition of cyclooxygenase-2 by various constituents (like flavonoids), (3) the release of cytotoxic H_2_O_2_ and (4) the scavenging of ROS and have been correlated with the phytochemical compounds [65]. Manuka honey has been shown to possess a potent anti-proliferative effect on murine melanoma (B16.F1), colorectal carcinoma (CT26) and human breast cancer (MCF-7) cell lines in a time- and dose-dependent manner [8]. The main mechanism by which it exerts such an anti-proliferative effect is through the activation of mitochondrial apoptotic pathways, involving the stimulation of the initiator, caspase-9, which determines the activation of the executioner, caspase-3 [65]. Moreover, it induces apoptosis via the activation of PARP, the induction of DNA fragmentation and the loss of Bcl-2 expression. *In vivo*, manuka honey is also effective in: (1) decreasing the tumor volume and increasing the apoptosis of tumor cells in a mouse melanoma model; and (2) reducing colonic inflammation in inflammatory bowel disease in rats, restoring lipid peroxidation and improving antioxidant parameters [65].

Finally, in healthy individuals, manuka honey UMF 20+ has been evaluated for its safety: its consumption showed: (1) no significant effect on the allergic status of the subjects; (2) no detrimental effect in relation to advanced glycation end products, which are implicated in a number of serious diseases, including renal disease, diabetes, neurodegenerative disease and heart disease; and (3) no change in gut microbiota homeostasis, confirming its safety for healthy individuals [66].

## 6. Conclusions

Besides its main components, manuka honey contains a large number of other constituents in small and trace amounts, able to exert numerous nutritional and biological effects, like antimicrobial and antioxidant activities. The above information shows that in microbiological and clinical tests, manuka honey offers advantages in controlling bacterial growth and in the treatment of several health problems. The easiness of administration in wound treatment and the absence of antibiotic resistance, which instead is found with conventional antibiotics, are important characteristics for the use of this honey in the treatment of clinical wounds.
